# Understanding the association between exposure to family planning messages and consistent condom use among never married men in Ghana

**DOI:** 10.1371/journal.pone.0255325

**Published:** 2021-08-24

**Authors:** Bright Opoku Ahinkorah, John Elvis Hagan, Abdul-Aziz Seidu, Eugene Budu, Georgina Yaa Mensah, Collins Adu, Thomas Schack

**Affiliations:** 1 Faculty of Health, School of Public Health, University of Technology Sydney, Sydney, Australia; 2 Department of Health, Physical Education, and Recreation, University of Cape Coast, Cape Coast, Ghana; 3 Faculty of Psychology and Sport Sciences, Neurocognition and Action-Biomechanics-Research Group, Bielefeld University, Bielefeld, Germany; 4 Department of Population and Health, University of Cape Coast, Cape Coast, Ghana; 5 College of Public Health, Medical and Veterinary Sciences, James Cook University, Douglas, Australia; 6 Department of Estate Management, Takoradi Technical University, Takoradi, Ghana; 7 Faculty of Health, Department of Communication Studies, University of Cape Coast, Cape Coast, Ghana; 8 Department of Health Promotion and Disability Studies, Kwame Nkrumah University of Science and Technology, Kumasi, Ghana; University of the Witwatersrand, SOUTH AFRICA

## Abstract

**Background:**

Despite considerable efforts to promote condom use, sexually active people in sub-Sahara Africa still engage in risky sexual behaviours, with condom use relatively low. With this high vulnerability of these persons to HIV and sexually transmitted infections, research related to exposure to family planning messages to help curb this trend remains sparse. This study examined how exposure to family planning messages in the midst of some socio-demographic factors is associated with consistent condom use among sexually active never married men in Ghana.

**Methods:**

Data were obtained from the 2014 Demographic and Health Survey (DHS) of Ghana. Only never married men (15–64 years) who have had sexual experience in the last 12 months were included in the analysis (N = 971). Frequencies, percentages, chi-square tests and binary logistic regression analyses were carried out. Results of the binary logistic regression analysis were presented using crude odds ratios (cOR) and adjusted odds ratios (aOR).

**Results:**

The results showed that only 26.15% of sexually active never married men in Ghana consistently used condom during sex. Men who were exposed to family planning messages were 51% more likely use condom consistently compared to those who are not exposed [aOR = 1.51, CI = 1.04–2.18]. In terms of the covariates, the likelihood of consistent condom use among men in Ghana was lower among those aged 35 years and above compared to those aged 15–24 [aOR = 0.46 CI = 0.21–0.99]. The odds of consistent condom use among men increased with level of education, with men with higher level of education having the highest odds of consistent condom use compared to those with no formal education [aOR = 9.98, CI = 2.05–48.46]. Men of the richest wealth quintile were more likely to use condom consistently compared to those of the poorest wealth quintile [aOR = 2.62, CI = 1.30–5.27]. Higher odds of consistent condom use was found among men who dwelled in the Central, Northern, and Upper East regions compared to those of the Western region.

**Conclusion:**

Our findings have established a strong association between exposure to family planning messages alongside age, educational level, wealth, and region of residence and consistent condom use. Men exposed to family planning messages were more likely to use condoms consistently. Designed programs should use intervention strategies that focus on interactive and participatory educational activities to improve sexually active men’s interpersonal communication on family planning messages, especially on consistent condom use with their sexual partners.

## Background

The past decade has seen a drastic social change that has swept away some longstanding customary norms and values related to suitable sexual practices across many societies, including sub-Saharan African countries [1–3]. Contemporary Ghana, like other sub-Saharan nations is now beset with high prevalence of risky sexual compromising practices such as unprotected sex, multiple sexual partnerships, premarital and extramarital affairs as well as increase early onset of sexual debut [4–7]. These diverse health compromising behaviours escalate the risk of sexually transmitted infections (STIs) and HIV infections not only among individuals involved with these high-risk sexual practices but among the general population as well [8–11]. For instance, unintended pregnancies are central to unsafe abortions; a major risk factor for maternal morbidity and mortality in sub-Saharan Africa (SSA) [12]. Another public health concern arising from sexual behaviours is the high prevalence of STIs, including HIV/AIDS among young people in general in SSA [13].

Globally, recent statistics show that family planning and/or contraceptive use prevented approximately 218 million unwanted pregnancies in 2012, stopping nearly 55 million unintended births, 138 million abortions, of which 40 million were unsafe, including 25 million miscarriages, and 118,000 maternal mortality [14]. Other reports revealed that compared to other regions (e.g., Oceania, 58%; Caribbean, Northern and Latin America, 75%), contraceptive use prevalence (36%) in SSA is low [15–18]. Hence, many health providers and promoters have employed diverse sexual and reproductive intervention programmes (e.g., family planning) aimed at empowering women and protecting them against risky sexual behaviours and the associated negative implications [16,19]. At the very focal point of this crusade is the engagement of men and their roles toward reproductive health services as a foundation for better improvement in the reproductive health of both men and women [20].

According to Shattuck et al. [21], men in developing societies, particularly in SSA are major decision-makers concerning family size and subsequent use of family planning. Available information reveals that a substantial disagreement between partners on family planning and desired family size has been reported together with low levels of communication on these topics [22–24]. The biggest challenge facing the quest to facilitate men’s involvement in family planning is the identification of appropriate information package that might efficiently boost their participation [24,25]. Importantly, inter-partner communication is reported to be associated with contraceptive decision-making that positively impacts contraceptive uptake and consistent use [26]. Conversely, refusing to communicate reproductive health goals restrict partners’ effective and consistent contraceptive use [23,24]. For example, partners who share information about safer sex or condom use are more likely to use condoms [27]. Also, individuals who negotiate with their sexual partners to use condoms are also more likely to use condoms than those who do not make such attempts [28].

With the advent of the AIDS epidemic, STIs, unmet need for family planning and high prevalence of unintended pregnancies in SSA, integral efforts to increase the practice of safer sex through the use of condoms have been suggested by major stakeholders and considered as very crucial towards intervention programmes and policy [29–32]. For most sexually active people, besides sex with an uninfected sexual partner, condom use is the only effective technique that guarantees protection against HIV and some other STIs [33,34]. Condom use has been proven as an effective method for preventing unwanted pregnancy and the most favoured method among unmarried sexually active people [35]. However, literature has reported a caveat on condom promotion and use for HIV/STIs prevention in low and middle-income countries where only consistent condom use has been found to offer effective protection [36]. According Hearst and Chen [36], the impact of condoms may often be moderated by inconsistent use, low use among persons at risk, and also show negative connections with other strategies (e.g., type of messages).

Previous studies on diverse ethnic young adults have identified a number of condom information sharing strategies such as risk communication (i.e., citing risks of unprotected sex), directives or requests, sex refusal without a condom, non-verbal introduction of condoms, emotional coercion (e.g., threatening negative consequences), seduction (e.g., using sexual arousal to distract partner), and deception (e.g., using false information) [28,37,38]. Other determinants often cited in literature to also moderate condom use range from type of partner, education, occupation, age, place of residence, multiple sexual partners, marital status, fertility preference, parity, and alcohol use [39–43]. Some of these factors have also been reported in SSA [44–48]. Surprisingly, none of these factors have consistently been established across these studies, but are quite hard to directly compare because of the diverse populations (e.g., commercial sex workers, clients, general population, and adolescents) used and at different time periods across different geographical locations.

In Ghana, like many patriarchal societies, sex and reproductive health communications between parents, adults and the young population are restricted to abstinence and menstrual hygiene, with minimal attention to condom use [49]. Given the vital role men play in the acceptance and utilization of contraceptives by partners, it is imperative and would be interesting to further investigate the connection between their exposure to family planning messages and consistent condom use among a cohort of never married men that previous research has ignored. Further, due to the high vulnerability of sexually active people to HIV/STIs and the risks of unintended pregnancy in SSA [30–32,50,51], it is for planned interventions and policy importance to better understand family planning messages and condom use behavior among sexually active men in order to help sexually active people lead healthy sexual and reproductive lives [45,52]. By increasing research on male involvement in reproductive health decision making, male partners can improve couple-level reproductive health outcomes by using condoms consistently with sexual partners to prevent HIV/STIs and unwanted pregnancies. Hence, the primary purpose of the current study was to examine how exposure to family planning messages in the midst of some socio-demographic factors is associated with consistent condom use among sexually active never married men in Ghana.

## Materials and methods

### Data source

Data for the study were obtained from the 2014 Demographic and Health Survey (DHS) of Ghana. DHS is a nationwide survey conducted every five-years across low- and middle-income countries. It focuses on maternal and child health by interviewing women of reproductive age (15–49 years) as well as men aged 15–64. DHS follows the same standard procedures: sampling, questionnaires, data collection, cleaning, coding, and analysis that allows for comparison between countries. To ensure national representativeness, the survey employs a two-stage stratified sampling technique [53]. The first stage consists of a list of primary sampling units (PSUs) or enumeration areas (EAs) that cover the entire country and are usually obtained from the latest national census when available. Each EA is further subdivided into standard size segments of about 100–500 households per segment. During this stage, a sample of predetermined segments is selected randomly with a probability proportional to the EA’s measure of size (i.e., number of households in an EA).The DHS survey personnel select households systematically from a list of previously enumerated households in each selected EA segment at the second stage. This is followed by in-person interviews in selected households. For the purpose of this study, only never married men (15–64 years) who had sexual experience in the last 12 months were included in the analysis (N = 971). The study excluded currently married, previously married and cohabiting men as they may have the intention to give birth and hence may have low likelihood of using condom.

### Measurement of variables

The outcome variable used was consistent condom use. To derive this variable, men were asked if they used condom every time they had sex with their 3^rd^ to most recent partner (1), 2^nd^ to most recent partner (2) and most recent partner (3) in the last twelve months. In each of these three occasions, the responses were ‘Yes’ and ‘No’. A composite variable was created from these three variables to derive the variable ‘consistent condom use’, which referred to men who always used condom on at least one of these occasions. This variable was coded as follows: No and Yes.

The key explanatory variable in this study was exposure to family planning messages. This was derived from three variables; heard of family planning on radio in the last few months, heard of family planning in newspaper/magazine in the last few months and heard family planning on television (TV) in the last few months. Each of these variables was coded as No and Yes. A composite variable was created from these three variables, with those who had either heard of family planning messages on radio, newspaper/magazine or TV in the last few months considered as those who had exposure to family planning messages (i.e., coded as Yes). Conversely, those who had neither heard of family planning messages on radio, newspaper/magazine nor TV in the last few months were considered as those who had no exposure to family planning messages (i.e., coded as No).

Apart from the key explanatory variable, other variables were included in the analysis as covariates. These variables were age, occupation, educational level, place of residence, religious affiliation, wealth quintile, marital status, and region. These variables were extracted from the males recode file of the 2014 DHS of Ghana. For ease of analysis, age, occupation, religious affiliation and marital status were recoded. Age was categorised into 15–24, 25–34, 35 years and above. Occupation was also categorised into two: not working and working. Religious affiliation was recoded as Christianity, Islam, and Other (Traditional religion and no religion). The choice of these explanatory variables was based on their availability in the dataset as well as their association with consistent condom use among men [54–56].

### Analytical procedure

Stata version 14.1 (College Station, TX) statistical analysis tool was used for the analysis. This software has the advantage of directly including robust standard errors that account for the complex sample design (i.e., two-stage sample design). Descriptive and inferential statistics were conducted. Descriptive analysis mainly focused on the presentation of frequencies and percentages for the socio-demographic characteristics of the respondents as well as the distribution of consistent condom use across exposure to family planning messages and socio-demographic characteristics of the respondents using chi-square test of independence. After this, a test for multicollinearity on the explanatory variables using the variance inflation factor (VIF) was carried out and the results showed no evidence of high collinearity (Mean VIF = 1.33, Maximum VIF = 2.21, and Minimum VIF = 1.03). The second stage involved a bivariate analysis of the independent association between the explanatory variables and consistent condom use. Finally, a multivariable analysis of the interaction between exposure to family planning and consistent condom use while controlling for the effect of the covariates was carried out. The results were reported as crude odds ratio (cOR) adjusted odds ratios (aORs) with 95% confidence intervals (CIs). All frequency distributions were weighted while the survey command (svy) in Stata was used to adjust for the complex sampling structure of the data in the regression analyses.

### Ethics approval

The men gave oral and written consent. The institutional review board (IRB) of the Ghana Health Service and ICF International institutional review board gave ethical approval for the survey. Permission to use the data set was requested from MEASURE DHS.

## Results

### Bivariate analysis on exposure to family planning messages, socio-demographic factors and consistent condom use

Results on exposure to family planning messages, socio-demographic factors and consistent condom use among sexually active never married men in Ghana are presented in Table 1. The results indicate that only 26.15% of sexually active never married men in Ghana consistently used condom during sex. Additionally, sexually active men in Ghana who consistently used condom were mostly those who are exposed to family planning messages (28.34%), those aged 25–34 (29.57%), those not working (30.11%), those with higher education (33.92%), urban dwellers (27.77%), Muslims (27.19%), those with richest wealth quintile (33.15%), and those from the Upper East region (37.68%). The Chi-square test showed that all the explanatory variables, except occupation, had statistically significant associations with consistent condom use among sexually active men in Ghana (see Table 1).

**Table 1:** Exposure to family planning messages, socio-demographic factors and consistent condom use among sexually active never married men in Ghana.

Variables			*Consistent condom use*	
	Weighted N	Weighted %	%	χ2 *(p-value)*
**Total**			26.15	
***Exposure to family planning messages***				7.63(p = 0.006)
No	256	26.40	20.06	
Yes	715	73.60	28.34	
***Age***				6.68(p = 0.035)
15–24	569	58.57	25.17	
25–34	336	34.60	29.57	
35 years and above	66	6.77	17.19	
***Occupation***				2.41(p = 0.120)
Not working	158	16.32	30.11	
Working	813	83.68	25.38	
***Educational level***				21.99(p<0.001)
No Education	30	3.10	7.54	
Primary	109	11.23	23.03	
Secondary	666	68.61	25.57	
Higher	166	17.06	33.92	
***Place of residence***				5.72(p = 0.017)
Urban	591	60.88	27.77	
Rural	380	39.12	23.63	
***Religious affiliation***				10.40(p = 0.006)
Christian	772	79.48	27.19	
Muslim	131	13.51	28.27	
Other	68	7.01	10.32	
***Wealth quintile***				15.09(p = 0.005)
Poorest	103	10.56	19.01	
Poorer	131	13.53	19.95	
Middle	204	21.04	24.92	
Richer	262	27.02	25.80	
Richest	270	27.85	33.15	
***Region***				25.73(p = 0.002)
Western	120	12.35	18.69	
Central	85	8.71	33.28	
Greater Accra	251	25.88	31.42	
Volta	66	6.76	20.94	
Eastern	89	9.12	27.05	
Ashanti	197	20.27	22.58	
Brong Ahafo	76	7.82	16.81	
Northern	53	5.51	33.77	
Upper East	17	1.79	37.68	
Upper West	17	1.79	28.16	

Source: Computed from 2014 GDHS.

### Bivariate and multivariable analysis on the influence of exposure to family planning messages and socio-demographic characteristics on consistent condom use

Table 2 presents results on the influence of exposure to family planning messages and socio-demographic characteristics on consistent condom use among sexually active never married men in Ghana. As shown in Table 2, men in Ghana who were exposed to family planning messages were more likely use condom consistently compared to those who were not exposed [aOR = 1.51, CI = 1.04–2.18]. In terms of the covariates, the likelihood of consistent condom use among men in Ghana was lower among those aged 35 years and above compared to those aged 15–24 [aOR = 0.46 CI = 0.21–0.99]. The odds of consistent condom use among men increased with level of education, with men with higher level of education having the highest odds of consistent condom use compared to those with no formal education [aOR = 9.98, CI = 2.05–48.46]. Men of the richest wealth quintile were more likely to use condom consistently compared to those with the poorest wealth quintile [aOR = 2.62, CI = 1.30–5.27]. Higher odds of consistent condom use was found among men who dwelled in the Central, Northern, and Upper East regions compared to those of the Western region (Model 2).

**Table 2:** Influence of exposure to family planning messages and socio-demographic characteristics on consistent condom use among sexually active men in Ghana.

Variable	Model 1	Model 2
	cOR (95% CI)	aOR (95% CI)
***Exposure to family planning messages***		
No	Ref	Ref
Yes	1.62** (1.15–2.29)	1.51* (1.04–2.18)
***Age***		
15–24	Ref	Ref
25–34	1.22 (0.90–1.65)	0.98 (0.69–1.40)
35 years and above	0.50 (0.25–1.00)	0.46* (0.21–0.99)
***Occupation***		
Not working	Ref	Ref
Working	0.75 (0.53–1.08)	0.98 (0.65–1.46)
***Educational level***		
No Education	Ref	Ref
Primary	4.96* (1.11–22.09)	6.66* (1.35–32.81)
Secondary	7.69** (1.84–32.15)	7.86** (1.69–36.55)
Higher	12.12**(2.82–52.13)	9.98**(2.05–48.46)
***Place of residence***		
Urban	Ref	Ref
Rural	0.70* (0.52–0.94)	1.22 (0.82–1.81)
***Religious affiliation***		
Christian	Ref	Ref
Muslim	1.33 (0.93–1.89)	1.46 (0.94–2.25)
Other	0.41* (0.21–0.820)	0.58 (0.28–1.18)
***Wealth quintile***		
Poorest	Ref	Ref
Poorer	0.93 (0.54–1.61)	1.14 (0.62–2.09)
Middle	1.37 (0.84–2.24)	1.65 (0.93–2.93)
Richer	1.45 (0.90–2.33)	1.73 (0.92–3.22)
Richest	2.07**(1.29–3.30)	2.62**(1.30–5.27)
***Region***		
Western	Ref	Ref
Central	2.61** (1.41–4.85)	2.72** (1.42–5.23)
Greater Accra	1.66 (0.94–2.95)	1.38 (0.76–2.51)
Volta	0.97 (0.47–2.00)	1.18 (0.57–2.46)
Eastern	1.42 (0.76–2.67)	1.51 (0.79–2.86)
Ashanti	1.18 (0.64–2.18)	0.95 (0.50–1.82)
Brong Ahafo	0.80 (0.41–1.57)	0.84 (0.42–1.67)
Northern	1.87 (0.97–3.59	2.75** (1.29–5.88)
Upper East	2.57**(1.32–5.01)	3.64**(1.68–7.89)
Upper West	1.66 (0.84–3.28)	1.53 (0.74–3.18)
N	971	971
Pseudo R^2^		0.168

Exponentiated coefficients; 95% confidence intervals in brackets.

* *p* < 0.05

** *p* < 0.01

*** *p* < 0.00.

cOR = crude odds ratio; aOR = adjusted odds ratio; CI = Confidence Interval; Ref = reference category.

## Discussion

The primary purpose of this study was to examine how exposure to family planning messages in the midst of some socio-demographic factors is associated with consistent condom use among sexually active never married men in Ghana. After controlling for confounders, the study showed that sexually active men in Ghana who were exposed to family planning messages were more likely to use condom consistently compared to those who were not exposed. Similar to this study, previous research [57] through a multicounty study in Kenya, Nigeria, and Senegal found that men who were exposed to family planning messages were more likely to use contraceptives (e.g., condom). This finding illuminates the critical importance associated with exposure to family planning issues through discussions on various media platforms. These exposures could improve men’s knowledge, and attitudes through behaviour modification that might translate to subsequent and regular use of condom. Some behaviours and communication change models emphasize that knowledge is one key prerequisite for a positive change in behaviour [58]. The possible reason for the finding could be that men who are exposed to family planning messages may understand the importance of consistent condom use and the risk associated with non-use of condom and will therefore develop a positive attitude towards its use.

Other results showed that consistent condom usage increased with level of education as well as wealth status with those in higher level of education and in the middle to the richest wealth quintile having higher odds of consistent condom use, a finding in line with previous research [57,59–61]. The possible reason for this finding could be that compared to men with no formal education, those with higher level of education are more exposed to media platforms and educational resources, discuss matters on HIV/STIs more often with better understanding of transmission modes, have less misconceptions about HIV/STIs, more positive attitudes toward condom use, and have positive perceptions of their personal sexual risk [62–64]. Formal education could also instill men with the capacity to better comprehend the family planning messages to practice safer sex, including condom use than their colleagues with little to no formal education [65]. Again, men with higher wealth status can deal with the financial barriers to access to contraceptives and hence can consistently buy and use condoms when required. This finding implies the need to intensify efforts to enhance the use of condom among men with no formal education and those with low wealth status through educational campaigns and other capacity building programmes.

Similar to previous research in SSA [57,66], a strong significant association between age and consistent condom use among men in Ghana was found in the current study. Specifically, as men aged, they were less likely to use condom, thus showing an inverse relationship between age and consistent condom use. Younger men, especially those not in steady relationships, and those with less numbers of lifetime sexual partners are more likely to use condoms consistently due to the fear of contracting HIV and STIs and becoming fathers through unintended pregnancies from their sexual partners [67,68]. Comparatively, the sexual behaviour and prevention forms of older men may be more resilient to changes as they age. According to Santelli et al. [69,70], the impact of age is most likely an image of the well-established global upsurge in condom use among young people.

Region of residence was found to be associated with the likelihood of consistent condom use among men in Ghana. Men in the Central, Northern, and Upper East were more likely to use condom consistently than to those in the Western Region. This finding corroborate previous studies [59,60,71] that found that contraceptive use vary by place of residence. In Ghana, Central, Northern and Upper East regions are considered among the regions where birth rate is very high [72]. The trend has been attributed to high rates of unintended pregnancies due to sociocultural norms within the regions that support polygamous marriages and large family sizes and inadequate access to contraceptives [51,73,74]. Hence, the higher likelihood of consistent condom use among never married men who live in those regions could be an intervention to reduce the high fertility rates in the region. Consequently, numerous interventions, campaigns and projects have been launched by both successive governments and non-governmental agencies to improve living conditions of those who live in those regions, including their decision making towards reproductive health. Therefore, it is possible that men’s behaviour in reproductive health issues is reflected by a mixture of both idiosyncratic characteristics and ideological differences that may promote or hinder consistent use of condom and perhaps other modern contraception.

### Strength and limitations

Some potential limitations inherent within the study are captured. First, the cross-sectional nature of the DHS shams the ability to establish causality but only associations between variables. Second, reporting odds ratios makes it difficult to understand the effect sizes of each independent variable on the outcome variable. Other limitations with the analysis may be related to the potential modifications in the coding of procedures in charts, tables and other data extraction errors. However, no substantial alterations were done during coding and extraction within the current study [72]. Another potential limitation is the measurement of exposure to mass media messaging- many men may get messages from sources other than those captured by the DHS, for example, through social media. Despite these, the relatively large sample size gave the study the statistical power to run rigorous analysis. The findings of this study is generalizable to populations in other countries that have similar characteristics like Ghana. Finally, identifying a positive association between exposure to family planning messages and consistent condom use through the use of binary logistic regression supports the accuracy of the analysis performed.

## Conclusion and implications

We have established a strong association between exposure to family planning messages and consistent condom use among sexually active never married men in Ghana, with those exposed to family planning messages more likely to use condoms consistently. We also found that age, educational level, wealth, religious affiliation, marital status, and region of residence are associated with consistent condom use among sexually active men in Ghana. Designed programs should use intervention strategies that focus on interactive and participatory educational activities to improve sexual active never married men’s interpersonal communication on family planning messages, especially consistent condom usage with their sexual partners. Findings of the current study emphasize the necessity for a better educational structure as part of the overall public health strategy to battle the AIDS and STIs epidemics and other negative outcomes through better decision-making skills.

## References

[1] Jamieson L., Milburn K. B., Simpson R., & Wasoff F. (2010). Fertility and social change: the neglected contribution of men’s approaches to becoming partners and parents. *The Sociological Review*, 58(3), 463–485. 10.1111/j.1467-954X.2010.01924.x.

[2] Ndulo M., & Grieco M. (Eds.). (2009). *Power*, *gender and social change in Africa*. Cambridge Scholars Publishing.

[3] Takyi, B. K. (2011, June). Transformations in the African family: A note on migration, HIV/AIDS and family poverty alleviation efforts in Sub‐Saharan Africa (SSA). In *United Nations Expert Group Meeting on Assessing Family Policies, New York* (pp. 1–3).

[4] Adedini SA, Mobolaji JW, Alabi M, Fatusi AO (2021) Changes in contraceptive and sexual behaviours among unmarried young people in Nigeria: Evidence from nationally representative surveys. PLoS ONE 16(2): e0246309. doi: 10.1371/journal.pone.0246309 33529246 PMC7853509

[5] Cortez R., Saadat S., Marinda E., and Odutolu O., Adolescent sexual and reproductive health in Nigeria (English). *Health*, *nutrition and population global practice knowledge brief* 2015, Washington, D.C.: World Bank Group.

[6] Doku D (2012). Substance use and risky sexual behaviours among sexually experienced Ghanaian youth. BMC Public Health 12(1): 1–7. doi: 10.1186/1471-2458-12-571 22839700 PMC3517501

[7] Paul M., Chalasani S., Light B., Knuttson A., and Fatusi A., Contraception for Adolescents and Youth: Being Responsive to their Sexual and Reproductive Health needs and Rights, in Issue Paper. 2019, UNFPA.

[8] Awusabo-Asare K., Bankole A., & Kumi-Kyereme A. Views of adults on adolescent sexual and reproductive health: Qualitative evidence from Ghana. *Occas Report*:2008:34.

[9] Pedrosa A. A., Pires R., Carvalho P., Canavarro M. C., & Dattilio F. Ecological contexts in adolescent pregnancy: the role of individual, sociodemographic, familial and relational variables in understanding risk of occurrence and adjustment patterns. *Contemporary Family Therapy*:2011:33(2):107.

[10] Sedgh G, Finer L. B., Bankole A., Eilers M. A., & Singh S. Adolescent pregnancy, birth, and abortion rates across countries: levels and recent trends. *Journal of Adolescents Health*:2015:56:223–30. doi: 10.1016/j.jadohealth.2014.09.007 25620306 PMC4852976

[11] World Health Organization. *WHO guidelines on preventing early pregnancy and poor reproductive health outcomes among adolescents in developing countries*. World Health Organization, Geneva.2011. 26180870

[12] Fako T. T. (2010). The connection between poverty, sexual activity, knowledge about HIV/AIDS and willingness to test for HIV infection among young people. *European Journal of social sciences*, 15(1), 115–128.

[13] UNICEF (2016). Strengthening the adolescent component of national HIV programmes through country assessments. Adolescent Assessment and Decision-Makers’Tool (AADM). Available from: http://data.unicef.org/wp-content/uploads/2016/04/Guidance-onCountry-Assessments-9_1_2015_252.pdf.

[14] WHO. Contraception. Fact sheet. 2014. Available from: https://apps.who.int/iris/bitstream/handle/10665/112319/WHO_RHR_14.07_eng.pdf?ua=1. [Accessed on august 24, 2020].

[15] Eliason S., Awoonor-Williams J. K., Eliason C., Novignon J., Nonvignon J., & Aikins M. (2014). Determinants of modern family planning use among women of reproductive age in the Nkwanta district of Ghana: a case–control study. *Reproductive health*, 11(1), 1–10. doi: 10.1186/1742-4755-11-1 25117887 PMC4274741

[16] Kalolo A., Mazalale J., Krumeich A. et al. Social cohesion, social trust, social participation and sexual behaviors of adolescents in rural Tanzania. *BMC Public Health* 19, 193 (2019). doi: 10.1186/s12889-019-6428-7 30764797 PMC6376705

[17] Nations United. Department of Economic and Social Affairs, Population Division (2017a): World Family Planning; 2017. Highlights (ST/ESA/SER.A/414). 2017.

[18] United Nations. Department of Economic and Social Affairs, population division (2017b). Model-based estimates and projections of family planning indicators. 2017. New York: United Nations; 2017.

[19] Sternberg P., & Hubley J. Evaluating men’s involvement as a strategy in sexual and reproductive health promotion. *Health Promotion International*:2004:19(3):389–396. doi: 10.1093/heapro/dah312 15306623

[20] Macro ORC. Malawi Demographic and Health Survey. Zomba, Malawi: National Statistical Office; 2005.

[21] Shattuck D., Kerner B., Gilles K., Hartmann M., Ng’ombe T., & Guest G. Encouraging contraceptive uptake by motivating men to communicate about family planning: the Malawi Male Motivator project. *American Journal of Public Health*:2011: 101(6): 1089–1095. doi: 10.2105/AJPH.2010.300091 21493931 PMC3093271

[22] Nzioka C. Research on men and its implications on policy and programme development in reproductive health. In: Programming for Male Involvement in Reproductive Health. Report of the Meeting of WHO Regional Advisors in Reproductive Health, WHO/PAHO, Washington DC, September 5–7, 2001. Geneva, Switzerland: World Health Organization:2002:143–152.

[23] Oyediran K. A., Ishola G. P., & Feyisetan B. J. (2002). Factors affecting ever-married men’s contraceptive knowledge and use in Nigeria. *Journal of Biosocial Science*:2002:34(4): 497–510. doi: 10.1017/s0021932002004972 12395865

[24] Paz Soldan V. A. How family planning ideas are spread within social groups in rural Malawi. *Studies in Family Planning*:2004:35(4): 275–290. doi: 10.1111/j.0039-3665.2004.00031.x 15628785

[25] Mistik S, Nacar M, Maziciolu M, Cetinkaya F. Married men’s opinions and involvement regarding family planning in rural areas. *Contraception*:2003:67(2): 133–137. doi: 10.1016/s0010-7824(02)00459-6 12586323

[26] Pulerwitz J, Barker G. Measuring attitudes toward gender norms among young men in Brazil: development and psychometric evaluation of the GEM Scale. *Men Masculinities*:2008;10(3): 322–338.

[27] Sheeran P, Abraham C, Orbell S. Psychosocial correlates of hetero-sexual condom use: A meta-analysis. Psychol Bull 1999;125:90–132. doi: 10.1037/0033-2909.125.1.90 9990846

[28] Bird ST, Harvey SM, Beckman LJ, et al. Getting your partner to use condoms: Interviews with men and women at risk of HIV/STDs. J Sex Res 2001;38:233–40.

[29] AIDSMark. A Decade of Innovative Marketing for Health: Lessons Learned. Washington DC: AIDSMark.2007.

[30] Awusabo-Asare K., Biddlecom A., Kumi-Kyereme A., & Patterson K. Adolescent sexual and reproductive health in Ghana: results from the 2004 National Survey of Adolescents. *Occasional Report*:2006:22.

[31] Hagan J. E., & Buxton C. Contraceptive knowledge, perceptions and use among adolescents in selected senior high schools in the central region of Ghana. *J Sociol Res*:2012:3(2):170–180.

[32] Krugu JK, Mevissen FEF, Debpuur C, & Ruiter RA. Psychosocial correlates of condom use intentions among junior high school students in the Bolgatanga municipality of Ghana. *International Journal of Sexual Health*:2016:28(1):96–110.

[33] Davis K. R., & Weller S. C. The effectiveness of condoms in reducing heterosexual transmission of HIV. *Family Planning Perspectives*:1999:272–279. 10614517

[34] Wald A., Zeh J., Selke S., Ashley R. L., & Corey L. Effect of condoms on reducing the transmission of herpes simplex virus type 2 from men to women. *JAMA*:2001: 285(24):3100–3106. [PubMed: 11427138]. doi: 10.1001/jama.285.24.3100 11427138

[35] Trussell J. Contraceptive efficacy. In: Hatcher R.; Trussell J.; Nelson AL.; Cates W.; Stewart FH.; Kowal D., editors. Contraceptive technology. 18. New York: Ardent Media:2003.

[36] Hearst N., & Chen S. Condom promotion for AIDS prevention in the developing world: is it working? *Studies in Family Planning*:2004:35(1):39–47. doi: 10.1111/j.1728-4465.2004.00004.x 15067787

[37] DeBro SC, Campbell SM, Peplau LA. Influencing a partner to use a condom: A college student perspective. Psychol Women Q 1994; 18:165–82. doi: 10.1111/j.1471-6402.1994.tb00449.x 12287707

[38] Edgar T, Freimuth VS, Hammond SL, et al. Strategic sexual communi-cation: Condom use resistance and response. Health Commun 1992;4: 83–104.

[39] Chingle M. P., Odunze P. A., Mohammed A., Bitto T. T., Sodipo O. Y., & Zoakah A. I. Predictors of male condom utilization in Plateau State, Nigeria. *Nigerian Journal of Clinical Practice*:2017:20(9):1079–1087. doi: 10.4103/njcp.njcp_56_17 29072229

[40] Ford K., & Norris A. E. Alcohol use, perceptions of the effects of alcohol use, and condom use in urban minority youth. *Journal of Acquired Immune Deficiency Syndromesand Human Retrovirology*: *Official Publication of the International Retrovirology Association*:1998:17(3):269–274. 10.1097/00042560-199803010-00013 9495228

[41] Long L., Yuan T., Wang M., Xu C., Yin J., Xiong C., et al. Factors associated with condom use among male college students in Wuhan, China. *PloS one*:2012:7(12). doi: 10.1371/journal.pone.0051782 23272167 PMC3525661

[42] Macaluso M., Demand M. J., Artz L. M., & Hook E. W. III Partner type and condom use. *Aids*:2000:14(5): 537–546. doi: 10.1097/00002030-200003310-00009 10780716

[43] Tschann J. M., Flores E., De Groat C. L., Deardorff J., & Wibbelsman C. J. Condom negotiation strategies and actual condom use among Latino youth. *Journal of Adolescent Health*:2010:47(3):254–262. doi: 10.1016/j.jadohealth.2010.01.018 20708564 PMC2923590

[44] Bankole A., & Malarcher S. Removing barriers to adolescents’ access to contraceptive information and services. *Studies in Family Planning*:2010:41(2):117–124. doi: 10.1111/j.1728-4465.2010.00232.x 21466111

[45] Bankole A., Ahmed F. H., Neema S., Ouedraogo C., & Konyani S. Knowledge of correct condom use and consistency of use among adolescents in four countries in Sub-Saharan Africa. *African Journal of Reproductive Health*:2007:11(3):197. 18458741 PMC2367135

[46] Lagarde E., Caraël M., Glynn J. R., Kanhonou L., Abega S. C., Kahindo M., et al. Educational level is associated with condom use within non-spousal partnerships in four cities of sub-Saharan Africa. *Aids*:2001:15(11):1399–1408. doi: 10.1097/00002030-200107270-00009 11504961

[47] Mnyika K. S., Klepp K. I., Kvale G., & Ole-King’Ori N. Determinants of high-risk sexual behaviour and condom use among adults in the Arusha region, Tanzania. *International Journal of STI & AIDS*:1997:8(3):176–183. doi: 10.1258/0956462971919840 9089028

[48] Oyediran K. A. Determinants of condom use among monogamous men in Ondo State, Nigeria. *Journal of Health*, *Population and Nutrition*:2003:358–366. 15038591

[49] Manu A. A., Mba C. J., Asare G. Q., Odoi-Agyarko K., & Asante R. K. O. (2015). Parent–child communication about sexual and reproductive health: Evidence from the Brong Ahafo region Ghana. Reproductive Health, 12(16), 1–13. doi: 10.1186/s12978-015-0003-1 25889521 PMC4359389

[50] Ahinkorah B. O., Hagan Junior J. E., Seidu A. A., Mintah J. K., Sambah F., Schack T., et al. Examining pregnancy related socio-cultural factors among adolescent girls in the Komenda-Edina-Eguafo-Abrem municipality in the central region of Ghana: a case-control study. *Frontiers in Public Health*:2019a: 7:93. doi: 10.3389/fpubh.2019.00093 31069207 PMC6491621

[51] Ahinkorah B. O., Hagan J. E. Jr, Seidu A. A., Budu E., Hormenu T., Mintah J. K., et al. Access to Adolescent Pregnancy Prevention Information and Services in Ghana: A Community-Based Case-Control Study. *Frontiers in Public Health*:2019b:7: 382. doi: 10.3389/fpubh.2019.00382 31921747 PMC6927296

[52] Manlove J., Ikramullah E., & Terry-Humen E. Condom use and consistency among male adolescents in the United States. *Journal of Adolescent Health*:2008:43(4):325–333. doi: 10.1016/j.jadohealth.2008.03.008 18809129

[53] Aliaga A., & Ren R. Optimal sample sizes for two-stage cluster sampling in demographic and health surveys. *Demographics and Health Research*:2006:30.

[54] Feldman B. S., Shtarkshall R. A., Ankol O. E., Sela T., & Kark J. D. Diminishing gender differences in condom use among a national sample of young Israeli men and women between 1993 and 2005. *Journal of Adolescent Health*:2012:50(3):311–314. doi: 10.1016/j.jadohealth.2011.05.014 22325139

[55] Hu L., Luo Y., Zhong X., Lu R., Wang Y., Sharma M., et al. Condom Use and Related Factors among Rural and Urban Men Who Have Sex With Men in Western China: Based on Information-Motivation-Behavioral Skills Model. *American Journal of Men’s Health*:2020:14(1):1557988319899799. doi: 10.1177/1557988319899799 32028826 PMC7008563

[56] Jin M., Yang Z., Dong Z., & Han J. Correlates of consistent condom use among men who have sex with men recruited through the Internet in Huzhou city: a cross-sectional survey. *BMC Public Health*:2013: 13(1):1101. doi: 10.1186/1471-2458-13-1101 24289100 PMC3912932

[57] Okigbo C. C., Speizer I. S., Corroon M., & Gueye A. Exposure to family planning messages and modern contraceptive use among men in urban Kenya, Nigeria, and Senegal: a cross-sectional study. *Reproductive Health*:2015:12(1):63. 26199068 10.1186/s12978-015-0056-1 PMC4508879

[58] Catania J. A., Keggles S. M., & Coates T. J. Towards understanding of risk behavior: An AIDS risk reduction model (AARM). *Health Educ Q*:1990:17(1):53–72. doi: 10.1177/109019819001700107 2318652

[59] Kabagenyi A., Ndugga P., Wandera S. O., & Kwagala B. Modern contraceptive use among sexually active men in Uganda: does discussion with a health worker matter?. *BMC Public Health*:2014:14(1):286. doi: 10.1186/1471-2458-14-286 24673890 PMC3986853

[60] Ochako R., Temmerman M., Mbondo M., & Askew I. Determinants of modern contraceptive use among sexually active men in Kenya. *Reproductive Health*:2017:14(1):56. doi: 10.1186/s12978-017-0316-3 28449723 PMC5408470

[61] Butame S. A. The prevalence of modern contraceptive use and its associated socio-economic factors in Ghana: evidence from a demographic and health survey of Ghanaian men. *Public Health*:2019:168: 128–136. doi: 10.1016/j.puhe.2018.12.020 30769244

[62] Blanc A. The Relationship Between Sexual Behaviour and Level of Education in Developing Countries. UNAIDS report: Key document. Geneva: UNAIDS.2000.

[63] Ingham R. AIDS: knowledge, awareness and attitudes. In: Sexual Behaviour and AIDS in the Developing World. Edited by Cleland J, Ferry B. London: Taylor & Francis:1995:43–74.

[64] Mehryar A. Condoms: Awareness, attitudes and use. In: Sexual Behaviour and AIDS in the Developing World. Edited by Cleland J, Ferry B. London: Taylor & Francis:1995:126–156.

[65] Gallant M., & Maticka-Tyndale E. School-based HIV prevention programmes for African youth. *Social Science & Medicine*:2004:58(7):1337–1351. doi: 10.1016/S0277-9536(03)00331-9 14759680

[66] Wilson H. W., Ameme D. K., & Ilesanmi O. S. Contraceptive Methods Accessed in Volta Region, Ghana, 2009–2014. *International Scholarly Research Notices*:2017. doi: 10.1155/2017/7257042 29082308 PMC5610863

[67] Kanda L., & Mash R. Reasons for inconsistent condom use by young adults in Mahalapye, Botswana. *African Journal of Primary Health Care & Family Medicine*:2018:10(1):1–7. doi: 10.4102/phcfm.v10i1.1492 29943592 PMC6018458

[68] Nasrullah M., Oraka E., Chavez P. R., Johnson C. H., & DiNenno E. Factors associated with condom use among sexually active US adults, national survey of family growth, 2006–2010 and 2011–2013. *The Journal of Sexual Medicine*:2017:14(4): 541–550. doi: 10.1016/j.jsxm.2017.02.015 28364979 PMC5477642

[69] Santelli J. S., Morrow B., Anderson J. E., & Lindberg L. D. Contraceptive use and pregnancy risk among U.S. high school students, 1991 e2003. *Perspectives on Sexual and Reproductive Health*:2006: 38:106–111. doi: 10.1363/psrh.38.106.06 16772192

[70] Santelli J. S., Lindberg L. D., Finer L. B., & Singh S. S. Explaining recent declines in adolescent pregnancy in the United States: the contribution of abstinence and improved contraceptive use. *American Journal of Public Health*:2007: 97:150–156. doi: 10.2105/AJPH.2006.089169 17138906 PMC1716232

[71] Asa S. S., Titilayo A., & Kupoluyi J. A. Assessment of Contraceptive Use by Marriage Type among Sexually Active Men in Nigeria. *International Quarterly of Community Health Education*:2018:38(3):181–194. doi: 10.1177/0272684X17749800 29307287

[72] Ghana Statistical Service (GSS) Ghana Health Service (GHS) and ICF Macro (2015). Ghana Health Service (GHS) and ICF Macro. Ghana Demographic and Health Survey 2014, GSS, GHS, and ICF Macro, Accra.

[73] Ahinkorah BO, Hagan JE Jr, Seidu AA, Mintah JK, Sambah F, Schack T, Hormenu T. Examining pregnancy related socio-cultural factors among adolescent girls in the Komenda-Edina-Eguafo-Abrem municipality in the central region of Ghana: a case-control study. Frontiers in public health. 2019 Apr 24;7:93. doi: 10.3389/fpubh.2019.00093 31069207 PMC6491621

[74] Seidu A. A., Hagan J. E., Agbemavi W., Ahinkorah B. O., Nartey E. B., Budu E., et al. Not just numbers: Beyond counting caesarean deliveries to understanding their determinants in Ghana using a population based cross-sectional study. *BMC Pregnancy and Childbirth*:2020:20(1):1–10. doi: 10.1186/s12884-020-2792-7 32070303 PMC7029601

